# Thoracoscopic surgery combined with endoscopic creation of a submucosal tunnel for a large complicated esophageal leiomyoma

**DOI:** 10.1186/s40792-020-00854-5

**Published:** 2020-05-06

**Authors:** Koki Oyama, Kenoki Ohuchida, Koji Shindo, Taiki Moriyama, Yoshitaka Hata, Masafumi Wada, Eikichi Ihara, Shuntaro Nagai, Takao Ohtsuka, Masafumi Nakamura

**Affiliations:** 1 Department of Surgery and Oncology, Graduate School of Medical Sciences, Kyushu University, 3-1-1 Maidashi, Higashi-ku, Fukuoka, 812-8582 Japan; 2 Department of Medicine and Bioregulatory Science, Graduate School of Medical Sciences, Kyushu University, Fukuoka, Japan; 3 Center for Advanced Medical Innovation, Kyushu University, Fukuoka, Japan; 4 Department of Diagnostic and Therapeutic Endoscopy, Kyushu University Hospital, Fukuoka, Japan; 5 Department of Gastroenterology and Metabolism, Graduate School of Medical Sciences, Kyushu University, Fukuoka, Japan

**Keywords:** Esophageal leiomyomas, Submucosal tunnel, Endoscopic and thoracoscopic procedures

## Abstract

**Background:**

The standard surgical method for symptomatic submucosal tumors (SMTs) or tumors with unclear biological behavior is enucleation. Minimally invasive approaches are usually considered appropriate for surgical enucleation; thus, thoracoscopic and laparoscopic enucleation is performed widely and safely. However, it is sometimes difficult to enucleate large and complicated esophageal tumors using thoracoscopic surgery, and even if rare, there is the risk of requiring thoracotomy or esophagectomy. In the present case, we enucleated a large and complicated leiomyoma safely using a new combined method with endoscopic and thoracoscopic procedures.

**Case presentation:**

A 42-year-old woman presented to our hospital for a detailed examination of an abnormal finding in her health check-up chest X-ray images. She complained of upper abdominal pain after eating, and computed tomography revealed an esophageal tumor measuring 60 mm in length surrounding her lower thoracic esophagus. Esophagogastroduodenoscopy revealed a huge complicated SMT at the esophagogastric junction. Cytological examination with endoscopic ultrasound-guided fine-needle aspiration showed that the tumor was a leiomyoma. To enucleate this large and complicated esophageal SMT safely and without damaging the esophageal mucosa, we performed endoscopic and thoracoscopic procedures. We created a submucosal tunnel, endoscopically, and then performed thoracoscopic surgery to enucleate the tumor completely from the esophageal muscularis. Using these combined procedures, we were able to easily mobilize even a complicated tumor of this size from the mucosa and completed the surgery thoracoscopically without difficulty. As a result, the tumor was dissected safely with a minimal defect in the muscularis and without damaging the mucosa. Finally, we closed the defect in the esophageal muscularis with continuous sutures, thoracoscopically, and closed the entry of the submucosal tunnel using clips, endoscopically.

**Conclusions:**

Using these combined procedures, we safely enucleated a huge complicated esophageal SMT. The increased mobility of the tumor after creating the submucosal tunnel contributed to the minimal defect in the muscular layer and prevented injury to the esophageal mucosa, possibly leading to fewer postoperative complications such as esophageal stenosis and local infection.

## Background

Esophageal leiomyomas, although rare, are the most common benign intramural tumor of the esophagus [1]. The incidence of esophageal benign tumors is relatively rare at 0.005–7.9% according to autopsy data, and leiomyomas account for 70–80% of these tumors [2]. Esophageal leiomyomas are typically found in patients between 20 and 50 years of age and are more frequent in men, with a ratio of men to women of 2:1 [3]. The most common complaints in symptomatic patients are epigastric discomfort (68%) and dysphagia (52%) [4], and the mean tumor diameter among symptomatic patients is 5.3 cm vs 1.5 cm in asymptomatic patients [4]. Therefore, generally, esophageal tumors smaller than 5 cm in diameter are asymptomatic. Most esophageal leiomyomas are located in the lower (53%) or middle (43%) thirds of the esophagus [4]. The standard surgical method for symptomatic submucosal tumors (SMTs) or tumors with unclear biological behavior is enucleation. Classically, thoracotomy was performed for this approach, but currently, minimally invasive approaches are considered appropriate for surgical enucleation of SMTs. Thoracoscopic and laparoscopic enucleation is performed widely and safely [5, 6], and recently, per oral endoscopic tumor resection (POET) has been developed as a more minimally invasive procedure for upper gastrointestinal SMTs [7]. However, the indications for POET are limited to tumors less than 40–50 mm in diameter because these tumors are removed through the mouth [7, 8]. Additionally, enucleating large and complicated tumors is sometimes difficult even with thoracoscopic surgery, and there is a risk of the procedure leading to thoracotomy or esophagectomy, although this is rare [9]. In the present case, we enucleated a large and complicated leiomyoma safely using a new combined method involving endoscopic and thoracoscopic procedures.

## Case presentation

**Fig. 1:**
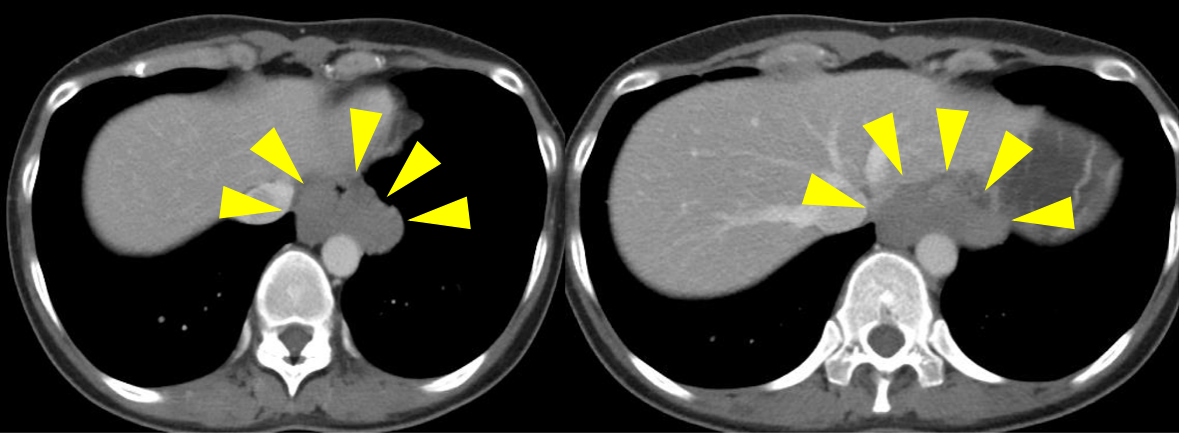
Computed tomographic images. A tumor surrounding the esophagus is seen at the lower thoracic esophagus measuring > 60 mm (major axis) with a complicated shape

**Fig. 2:**
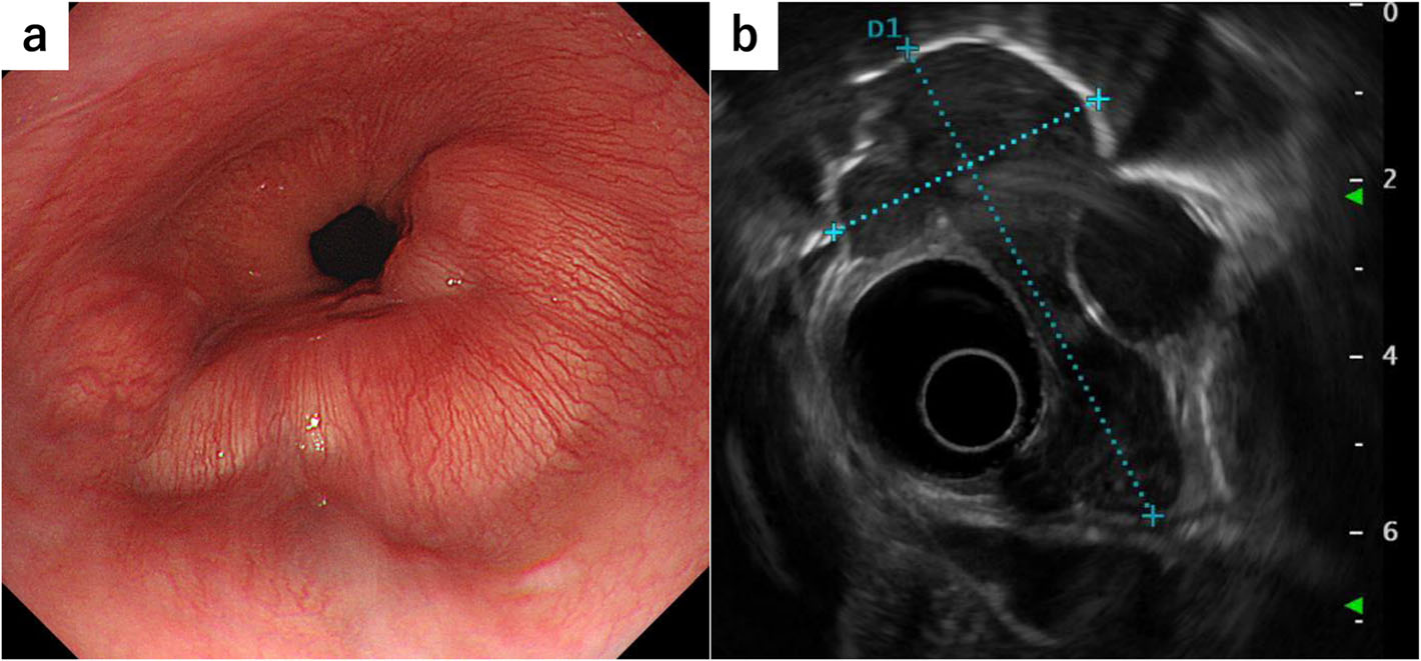
Endoscopic findings. **a** Submucosal tumor with a sub-circumferential elevated lesion near the esophagogastric junction. **b** Endoscopic ultrasonographic image showing the irregular-shaped tumor arising from the muscularis

**Fig. 3:**
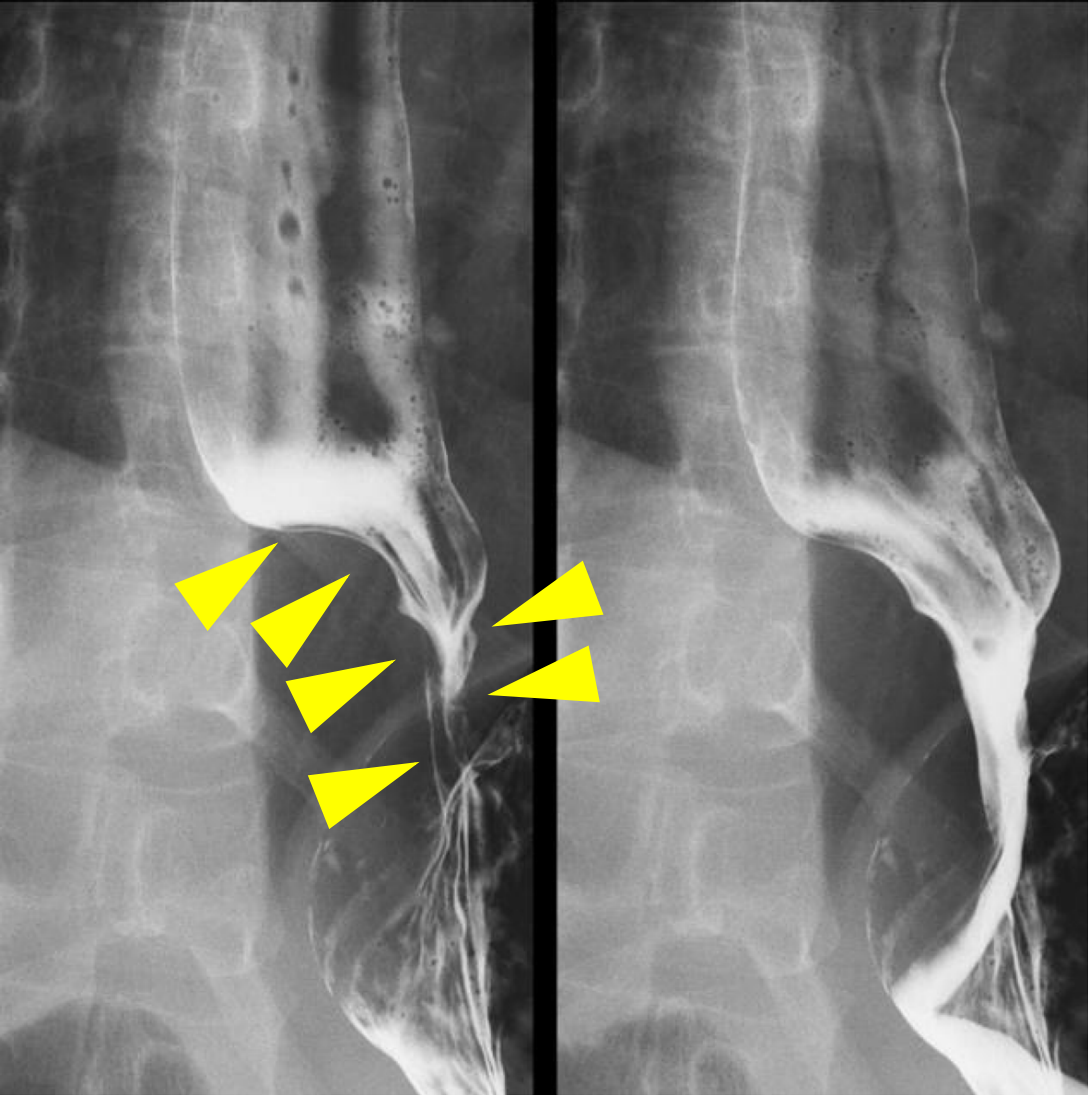
Esophagography. A 40-mm stenosis owing to the tumor is seen in the lower thoracic esophagus

A 42-year-old woman presented to our hospital for detailed examination of an abnormal shadow on her health check-up chest X-ray images. She complained of upper abdominal pain after eating, for more than 6 months. She had no remarkable medical history, and her general status was good. She was initially evaluated with computed tomography (CT), which revealed a tumor surrounding the lower thoracic esophagus. The tumor measured > 60 mm (major axis) and was slightly enhanced in the delayed phase with enhanced CT, and the shape of the tumor was extremely complicated (Fig. 1). She underwent esophagogastroduodenoscopy, which revealed an SMT with a sub-circumferential elevated lesion near the esophagogastric junction (Fig. 2a). Endoscopic ultrasonography (EUS) revealed an irregular-shaped tumor arising from the muscularis, which surrounded the lower esophagus for 3/4 of its circumference (Fig. 2b). EUS-guided fine-needle aspiration (EUS-FNA) biopsy indicated a leiomyoma. Esophagography revealed a 40-mm stenosis owing to the tumor, in the lower thoracic esophagus (Fig. 3). Considering the tumor size and its complicated shape, we planned to perform a hybrid operation with endoscopic and thoracoscopic procedures to enucleate the tumor safely without injuring the mucosa and with a minimal defect in the esophageal muscularis.

### Surgical procedure

**Fig. 4:**
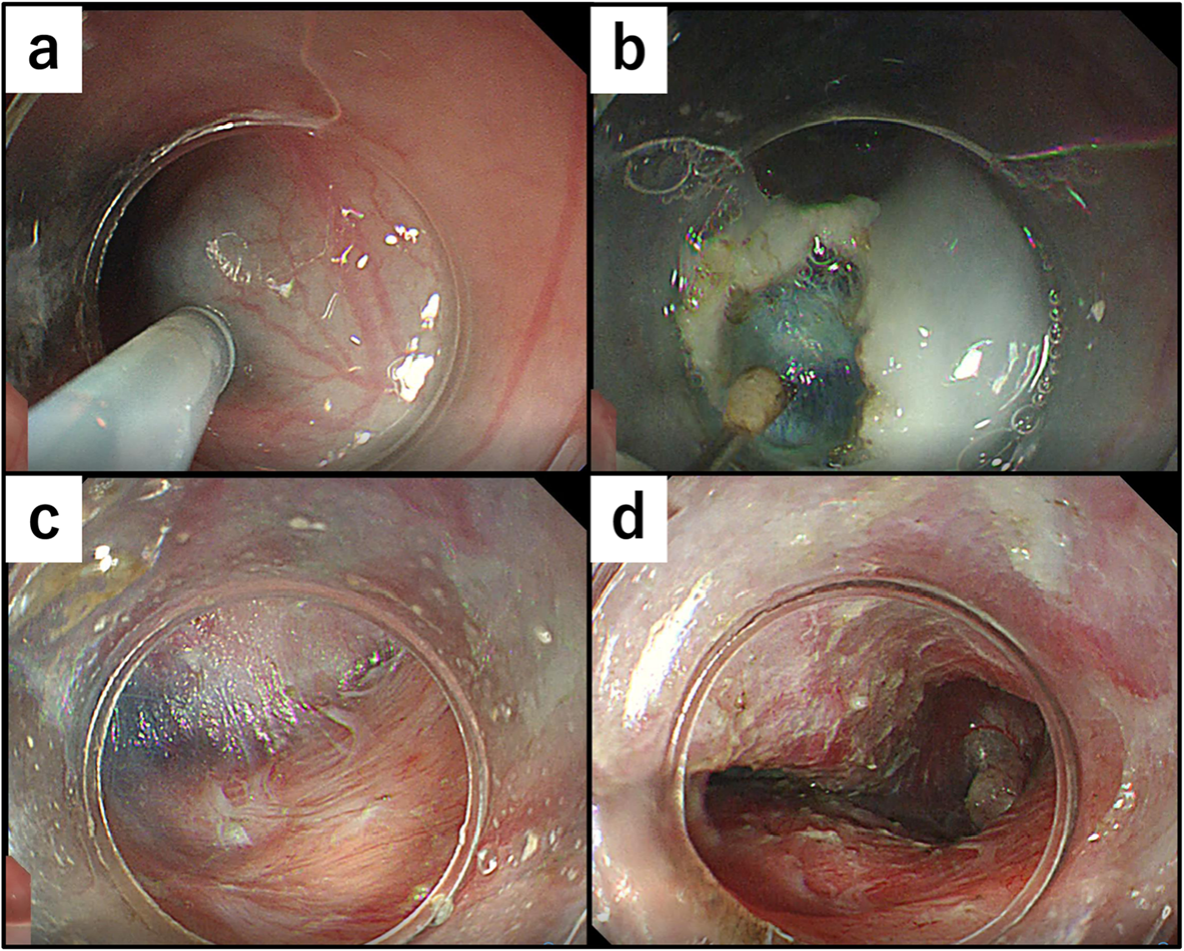
Endoscopic surgical procedure. **a** Injecting indigo carmine into the submucosal layer. **b** Creating the entry to the submucosal tunnel 30-mm proximal to the oral edge of the tumor. **c** Dissecting the layer between the mucosal and muscular layers. **d** Extending the submucosal tunnel beyond the anal edge of the tumor and separating the tumor from the mucosa

**Fig. 5:**
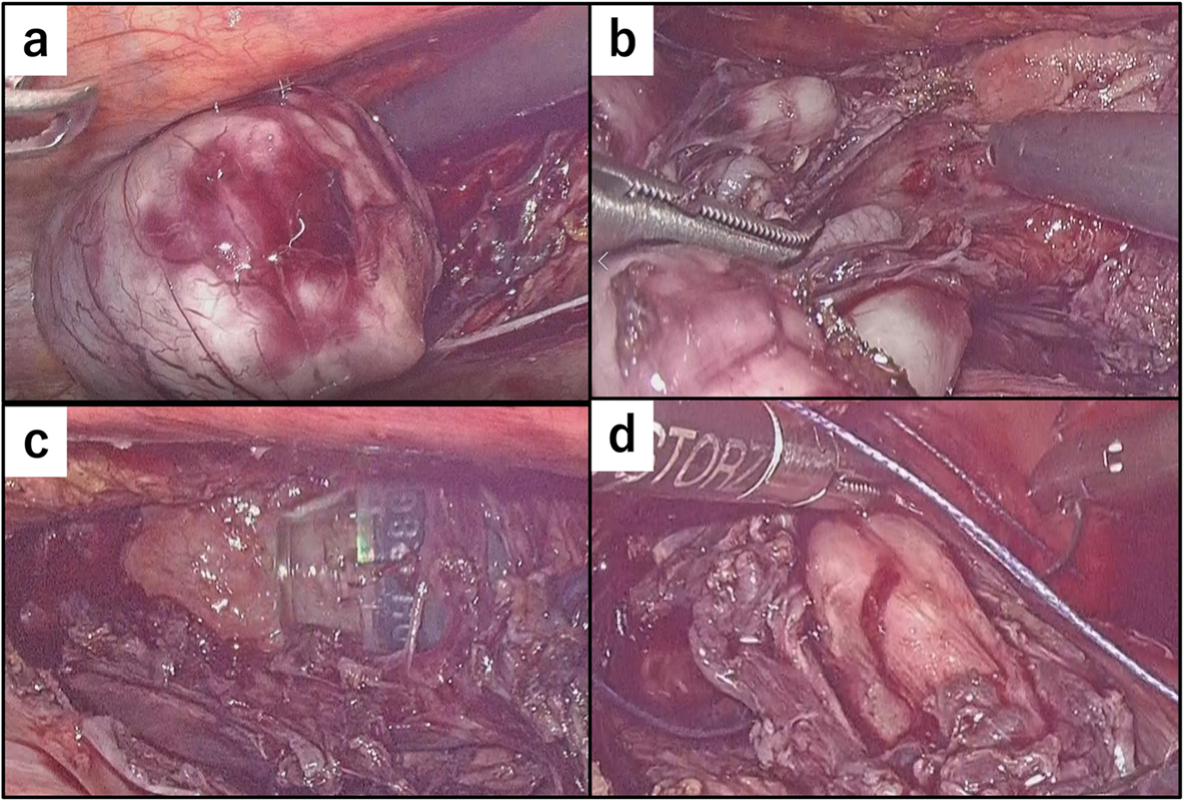
Thoracoscopic surgical procedure. **a** Cutting the outer membrane of the esophagus around the tumor then dissecting the muscular layer in the esophageal wall along the cut edge of the outer membrane. **b** Connecting the enucleation layer to the submucosal tunnel created during the endoscopic procedure. **c** View after enucleating the tumor with the endoscope passed through the submucosal tunnel. **d** Closing the defect in the muscularis using continuous sutures

**Fig. 6:**
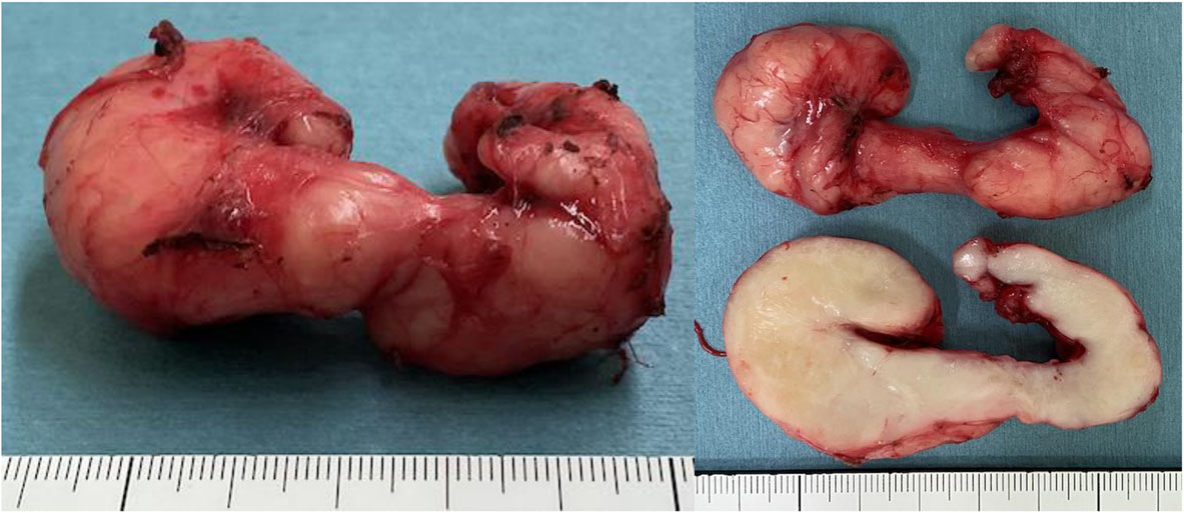
The resected specimen. The tumor was resected completely without injuring the surrounding structures. The image shows the tumor’s irregular and complicated shape

The patient underwent general anesthesia, and we first performed the endoscopic procedure with the patient in the supine position. We injected indigo carmine into the submucosal layer to create a space between the mucosa and muscularis (Fig. 4a); then, we created the entry of the submucosal tunnel 30-mm proximal to the oral edge of the tumor (Fig. 4b). Next, we dissected the blue-stained layer between the mucosa and muscularis using an electrocautery knife (triangle-tip knife) (KD-640L, Olympus, Tokyo, Japan) and extended the submucosal tunnel anally and laterally (Fig. 4c). We then extended the submucosal tunnel beyond the anal edge of the tumor and separated the tumor from the mucosa (Fig. 4d). Next, we changed the patient’s position from supine to left hemi-lateral for the thoracoscopic procedure, which we performed under 10 mmHg to provide artificial pneumothorax. We placed the following four ports: a 12-mm port at the posterior axillary line in the 7th intercostal space (ICS), a 12-mm port at the posterior axillary line in the 9th ICS, a 5-mm port at the mid-axillary line in the 5th ICS, and a 5-mm port at the anterior axillary line in the 8th ICS. After opening the mediastinal pleura longitudinally along with the esophagus, we identified the tumor at the lower posterior mediastinum and began the enucleation. First, we cut the outer membrane of the esophagus around the tumor, then dissected the muscularis along the tumor to connect the layer of enucleation with the endoscopic submucosal tunnel (Fig. 5a, b). Despite its shape and size, we handled the tumor easily because the tumor was mobilized from the mucosa endoscopically when we created the submucosal tunnel. We completed the enucleation without damaging the mucosa and with minimal defect in the muscularis (Fig. 5c). The dissected tumor was extracted through the expanded working port. We closed the defect in the muscularis thoracoscopically with continuous 4-0 PDS (Ethicon Inc., Somerville, NJ) sutures and closed the entry of the submucosal tunnel with clips, endoscopically (Fig. 5d). Finally, we placed a 6.5-mm multichannel drain through the 12-mm port in the 9th ICS. The operation time was 273 min (endoscopic procedure: 68 min, thoracoscopic procedure: 185 min), and the blood loss amount was 25 g. The tumor was resected completely without injuring the surrounding structures, and the tumor had an irregular and complicated shape, as in preoperative images (Fig. 6). We performed esophageal endoscopy on postoperative day 2 and confirmed that there was no leakage or stenosis. Oral intake was started on postoperative day 3, and the patient was discharged on postoperative day 7 with no adverse events.

## Discussion

Esophageal leiomyomas are the most common benign intramural tumors of the esophagus. The frequency of esophageal leiomyoma is approximately 1/50 that of esophageal carcinoma [10]. Leiomyomas grow slowly and are usually asymptomatic, so they may be discovered incidentally. According to the 40-year database of the Massachusetts General Hospital [4], mean tumor diameter among symptomatic patients was 5.3 cm compared with 1.5 cm in asymptomatic patients. Frequent symptoms are epigastric distress, namely, heartburn and other epigastric symptoms commonly attributed to gastroesophageal reflux, and dysphagia. Esophageal endoscopy, chest magnetic resonance imaging, CT, EUS, and EUS-FNA are useful to diagnose esophageal SMTs. We did not perform positron-emission tomography/CT (PET/CT) because EUS-FNA indicated a leiomyoma. Previous reports stated that the diagnostic accuracy of EUS-FNA was 86–100% for upper gastrointestinal SMT and that the sensitivity and specificity of preoperative EUS-FNA in surgical cases of gastrointestinal stromal tumor (GIST) were 82–100% and 100%, respectively [11–13]. Additionally, there are several reported cases of esophageal leiomyoma with false-positive findings using PET/CT, suggesting that esophageal leiomyomas may have a wide spectrum of fluorine-18-fluorodeoxyglucose (FDG) uptake [14, 15]. Therefore, we do not perform PET routinely for patients with SMT, when we obtain sufficient EUS-FNA samples. However, one study reported that the diagnostic yield of EUS-FNA for upper gastrointestinal SMT ranged widely from 64 to 93% [16]. Baysal et al. reported that samples derived from EUS-FNA were considered insufficient or nondiagnostic in 48% of cases of esophageal leiomyoma [17]. Therefore, in the present study involving a large and complicated tumor, PET-CT should have been performed to confirm no malignancy.

In many patients, SMTs are not definitively diagnosed until after resection and subsequent pathological examinations. Thus, surgical resection is recommended for tumor diagnosis and treatment in patients with tumors larger than 3 cm, in symptomatic patients, and in those with findings of potential malignancy [18]. Generally, thoracoscopic enucleation is the recommended surgical treatment for tumors measuring 1–5 cm in diameter [19]. Normally, enucleation is the chosen treatment for benign tumors that are symptomatic or rapidly increasing in size. Although Robb et al. reported that enucleation for esophageal GIST was oncologically safe for tumors less than 65 mm in diameter [20], esophagectomy should be selected instead of enucleation to ensure curability, if there is a possibility of malignancy, as reported previously [21]. However, there are remarkable differences in postoperative quality of life between enucleation and esophagectomy; therefore, clinically, it is quite difficult to choose it for the patients who have the tumors with malignant potential although we usually decide it with sufficient informed consent to patients. Further studies are necessary to evaluate whether enucleation is appropriate in patients with low-level malignant disease such as esophageal GIST. Previously, the classical approach for enucleation was thoracotomy; however, video-assisted thoracoscopic surgery has replaced the traditional procedure because the minimally invasive video-assisted procedure is associated with fewer pulmonary complications, shorter hospital stays, and less postoperative wound-related pain compared with open surgery [22–24]. Recently, POET has been reported as a new surgical technique, which involves creating a submucosal tunnel endoscopically to remove the tumor [7, 25]. This procedure allows for complete endoscopic resection and contributes to earlier start of postoperative oral intake, shorter hospital stays, and fewer short-term postoperative complications. However, POET is associated with relatively high risks of surgical complications and technical difficulties in patients with tumors larger than 3 cm in diameter, or tumors with a complicated shape [26]. Therefore, POET was not appropriate in our patient with a large and complicated tumor.

A laparoscopic approach may be possible for esophageal SMTs located at the junction; however, in our patient, because of the size of the tumor and its irregular shape, we required more space to completely and safely resect the tumor. The advantage of a thoracoscopic approach is that this approach provides sufficient working space, and we can rotate and move the mobilized tumor around the esophagus, with a large working space. With large and complicated tumors, a laparoscopic approach is more difficult because we mobilize the tumor from the oral side of the tumor to rotate it into the intraperitoneal cavity. However, in patients with pulmonary dysfunction, if it is difficult to perform differential lung ventilation, a laparoscopic approach is more suitable than a thoracoscopic approach. In the present case, pulmonary function was normal, and the tumor was located mainly in the lower thoracic esophagus. Therefore, we chose a thoracoscopic approach.

In our patient, preoperative imaging revealed that the tumor had a diameter > 60 mm, an irregular shape, and surrounded the esophagus for 3/4 of the esophageal circumference. We were concerned about the potential risks of accidental mucosal injury to the esophagus or creating a large defect in the muscularis if we performed conventional thoracoscopic surgery to enucleate the tumor. Furthermore, even if rare, there was the possibility of requiring conversion to esophagectomy or thoracotomy during the surgery. In most cases, we can perform enucleation for esophageal SMTs safely with conventional thoracoscopic surgery without complications. However, we experienced a case in which we had to convert enucleation to esophagectomy because of the large size and complicated shape of the tumor. Therefore, to enucleate the tumor easily and safely without injuring the mucosa and with a minimal defect in the esophageal muscularis, we performed the endoscopic procedures used in POET and created a submucosal tunnel to separate the tumor from the mucosa. The endoscopic creation of the submucosal tunnel increased the tumor’s mobility, which made it easier to handle the tumor safely and without difficulty during thoracoscopic enucleation.

We selected the left hemi-lateral position for the thoracoscopic procedure because this position allows concurrent endoscopic and thoracoscopic approaches. Other reasons that we chose this position are that the tumor was located mainly on the right side of the esophagus and that we were accustomed to approaching from the right side in thoracoscopic esophagectomy for esophageal cancer.

As a result, the tumor was completely enucleated without injuring the mucosa and with a minimal defect in the esophageal muscularis. However, if the tumor is too close to the esophageal orifice, it may be impossible to create a mucosal incision as the entry to the submucosal tunnel, and it may be difficult to perform a stable endoscopic procedure. The procedure is not difficult for GISTs or schwannomas; however, in the tumors with delle or tumors after boring biopsy, it may be difficult to create a submucosal tunnel without damaging the capsule or mucosa. These combined procedures are a promising treatment, even for malignancies such as gastrointestinal stromal tumors, although further investigation is needed to evaluate the oncological safety of our approach.

## Conclusion

Our procedures permitted enucleation of a large complicated esophageal SMT, which are associated with the risks of requiring esophagectomy or thoracotomy, although this is rare. Furthermore, our procedures resulted in a minimal defect in the esophageal muscularis and prevented esophageal injury, possibly leading to fewer postoperative complications such as esophageal stenosis and local infection. Our combined thoracoscopic and endoscopic procedures are a useful option for large and complicated esophageal SMTs.

## Data Availability

Not applicable.

## Abbreviations

EUS: Endoscopic ultrasound
CT: Computed tomography
POET: Per oral endoscopic tumor resection
ICS: Intercostal space
SMT: Submucosal tumor
FNA: Fine-needle aspiration
PET: Positron-emission tomography
GIST: Gastrointestinal stromal tumor
